# Advances in Functional Genomics of Disease Resistance in Cucumber (*Cucumis sativus*) and Translational Prospects for the Cucurbitaceae Family

**DOI:** 10.3390/genes17050522

**Published:** 2026-04-29

**Authors:** Zhipeng Wang, Fanqi Gao, Guangchao Yu

**Affiliations:** 1 College of Chemistry and Life Sciences, Anshan Normal University, Anshan 114007, China; wangzp1326@gmail.com (Z.W.); 13079219950@163.com (F.G.); 2 Liaoning Key Laboratory of Development and Utilization for Natural Products Active Molecules, Anshan Normal University, Anshan 114007, China

**Keywords:** cucumber, powdery mildew, downy mildew, target spot disease, disease resistance genes, multi-omics

## Abstract

Cucurbit crops—including cucumber (*Cucumis sativus*), watermelon (*Citrullus lanatus*), and melon (*Cucumis melo*)—are of major economic and nutritional importance worldwide. Yet their productivity and quality are severely compromised by foliar fungal diseases, particularly powdery mildew (PM), downy mildew (DM), and target leaf spot (TLS). While PM and DM have been extensively studied, TLS has emerged as an increasingly prevalent and damaging disease in key production regions, yet it remains comparatively understudied—especially with respect to its molecular basis and comparative pathobiology relative to PM and DM. Current reliance on chemical fungicides is hampered by escalating pathogen resistance and concerns over residual toxicity, whereas conventional breeding approaches face inherent limitations in pyramiding durable, broad-spectrum resistance against multiple pathogens. In this context, cucumber has emerged as a pivotal model species for dissecting foliar disease resistance mechanisms in cucurbits, supported by a high-quality reference genome, extensive resequencing datasets, diverse germplasm collections, and an efficient Agrobacterium-mediated transformation system. Despite these advantages, existing reviews predominantly address PM or DM resistance in isolation; comprehensive syntheses integrating TLS resistance advances—and critically, cross-disease comparisons of genetic architecture, transcriptional reprogramming, and defense signaling—are notably scarce. Furthermore, the translational pipeline—from gene discovery and functional validation to deployment in marker-assisted or genome-edited breeding—lacks systematic evaluation. Here, we provide a focused, cucumber-centered review that (i) synthesizes recent progress in mapping QTLs and GWAS loci, and characterizing key resistance-associated gene families (such as *NLRs*, *RLKs*, *PR* genes) conferring resistance to PM, DM, and TLS; (ii) integrates transcriptomic, epigenomic, and proteomic evidence to delineate conserved versus pathogen-specific host responses; (iii) highlights breakthroughs and unresolved questions in TLS resistance research, including the roles of novel susceptibility factors and non-canonical immune regulators; and (iv) critically assesses bottlenecks in translating resistance genes into practical breeding outcomes—such as linkage drag, functional redundancy, and genotype-by-environment interactions—and proposes empirically grounded strategies for accelerating molecular design of multi-disease-resistant cultivars. Collectively, this review aims to bridge fundamental insights with applied breeding goals, offering a conceptual and strategic framework for integrated management of foliar fungal diseases and the development of durable, broad-spectrum resistance in cucurbits.

## 1. Introduction

Cucurbit crops—including cucumber (*C. sativus*), watermelon (*C. lanatus*), melon (*C melo*), and pumpkin (*Cucurbita* spp.)—are globally vital for food security, nutrition, and agricultural economies. According to FAO FAOSTAT (2022), global production of cucumbers and gherkins, watermelons, and melons plus other non-watermelon cucurbits totaled 223 million metric tons: cucumbers and gherkins contributed 94.7 million tons, watermelons 100.0 million tons, and melons and related cucurbits 28.6 million tons [1]. Beyond their economic value, these crops are rich dietary sources of vitamins (such as vitamin C, provitamin A), essential minerals (such as potassium, magnesium), and health-promoting phytochemicals (such as cucurbitacins, carotenoids), underpinning their critical roles in fresh consumption, processing industries, export markets, and diversified human nutrition. Ensuring their stable, high-yielding, and high-quality production is thus indispensable to global vegetable supply chains and nutritional resilience.

Yet their productivity is increasingly undermined by three devastating foliar fungal diseases: powdery mildew (PM), downy mildew (DM), and target leaf spot (TLS). PM, caused by *Podosphaera xanthii*, induces severe yield losses (10–40%) across diverse agroecosystems [2]; DM, caused by *Pseudoperonospora cubensis*, is widely regarded as the most destructive disease of cucumber—earning the moniker “cucumber cancer”—and routinely causes 20–60% yield reductions under epidemic conditions [3]; TLS, caused by *Corynespora cassiicola*, has surged in incidence and severity in protected cultivation systems across Asia, with documented field losses ranging from 15% to 50% [4]. Chemical fungicides remain the primary control strategy; however, widespread pathogen resistance and regulatory restrictions on residue levels have eroded their long-term viability [5]. Conventional breeding, while foundational, faces fundamental constraints: lengthy development cycles (8–12 years), susceptibility to resistance breakdown due to pathogen evolution, and particular difficulty in stacking durable, broad-spectrum resistance against multiple pathogens simultaneously. Critically, despite its escalating agronomic impact, TLS lags far behind PM and DM in genetic and molecular research investment—resulting in a pronounced knowledge gap in its resistance mechanisms and host–pathogen interactions.

Advances in high-throughput sequencing and functional genomics now offer transformative tools for accelerating disease resistance research. Genome sequencing, pan-genome construction, and integrative multi-omics approaches—including transcriptomics, proteomics, metabolomics, and epigenomics—enable systematic, genome-wide identification of resistance loci, dissection of complex quantitative resistance architectures, and prioritization of functionally validated targets for molecular breeding [6]. Over the past three decades, foundational work—including cloning and functional characterization of canonical NLR resistance genes, MLO susceptibility factors, and key immune regulators (such as MAPK cascades, WRKY transcription factors)—has established the genomic framework for cucurbit disease resistance [7]. Since the 2010s, network-based multi-omics integration has become central to deciphering context-dependent defense signaling; more recently, systems biology modeling and AI-driven data mining have enabled the extraction of predictive regulatory modules and causal pathways from large-scale omics datasets [8].

Cucumber stands out as the preeminent model species for functional genomics of foliar disease resistance in the Cucurbitaceae. Its reference genome was first published in 2009, and subsequent refinements—including chromosome-level assemblies, pangenome resources, and extensive resequencing of diverse accessions—have yielded a highly annotated, approximately 367 Mb genome encoding approximately 26,000 high-confidence genes [3]. Robust, reproducible Agrobacterium-mediated transformation protocols (with efficiencies of 5–15% in elite lines) and *CRISPR*/*Cas9* editing systems are well established [9]. Complementing these tools is a genetically diverse core germplasm collection comprising thousands of accessions—enabling high-resolution QTL mapping, genome-wide association studies (GWAS), and allele mining across natural and induced variation [6]. Collectively, these resources provide an unparalleled platform for dissecting the genetic and molecular basis of multi-disease resistance to PM, DM, and TLS.

Despite substantial progress in elucidating powdery mildew (PM) and downy mildew (DM) resistance in cucumber, the current literature remains fragmented, as most existing reviews address single diseases in isolation and provide limited cross-disease synthesis, particularly for target leaf spot (TLS), whose genetic and molecular basis remains comparatively underexplored. Consequently, important questions remain insufficiently resolved, including which resistance components are shared versus pathogen-specific across PM, DM, and TLS, and to what extent currently reported loci and genes are truly supported for translational deployment in marker-assisted selection or genome editing rather than remaining preliminary candidates. To address these gaps, this review uses cucumber as a focal model to synthesize recent advances in QTL/GWAS mapping, resistance-related gene families, and multi-omics studies associated with these three major foliar diseases, with particular attention to the emerging yet still incomplete TLS research landscape; it further evaluates the current evidence supporting the translation of gene discovery into breeding application and briefly discusses how insights gained from cucumber may inform resistance improvement in other major cucurbit crops.

## 2. Identification and Characterization of Disease Resistance Gene Resources

Cucumber resistance to powdery mildew (PM), downy mildew (DM), and target leaf spot (TLS) is predominantly quantitative—governed by multiple QTLs whose effects are modulated by genetic background, pathogen race diversity, and environmental conditions. While recent studies have identified numerous resistance-associated QTLs—providing foundational resources for mechanistic dissection and molecular breeding—their translational utility remains constrained by inconsistent reproducibility across populations, variable mapping resolution (often >1–5 Mb), and limited functional validation. This gap has resulted in a proliferation of statistical signals without corresponding causal genes or robust markers—a hallmark “signal-rich but target-poor” landscape. To bridge this gap, this chapter adopts stability and translational readiness as unifying criteria. We first synthesize high-confidence, repeatedly validated QTL hotspots across the three diseases and their associated candidate gene leads; second, we evaluate the complementary power of genome-wide association studies (GWAS) in natural germplasm for resolving complex resistance architectures; third, we outline integrative strategies that synergize QTL and GWAS to converge statistical signals into narrow, functionally interpretable intervals; and we first synthesize high-confidence, repeatedly validated QTL hotspots across the three diseases and their associated candidate gene leads; second, we evaluate the complementary power of genome-wide association studies (GWAS) in natural germplasm for resolving complex resistance architectures; and third, we outline integrative strategies that combine QTL and GWAS evidence to prioritize narrower, functionally interpretable regions.

### 2.1. QTL Mapping Studies

Collectively, these studies indicate that foliar disease resistance is largely quantitative and influenced by genetic background, pathogen diversity, and environmental conditions. The resulting QTL landscape is extensive but uneven: some loci have been repeatedly detected across populations and validated by fine mapping or functional studies, while others remain preliminary signals in single biparental crosses. In the following subsections, we prioritize the former, focusing on major-effect loci, consistently identified genomic regions, and intervals with plausible candidate support.

#### 2.1.1. QTLs for Powdery Mildew Resistance

Powdery mildew (PM), primarily caused by *P. xanthii*, is one of the most widespread foliar diseases in cucumber production worldwide [10,11]. In cucumber, resistance to PM is typically quantitative, showing continuous phenotypic variation and moderate-to-high heritability [12,13]. Although monogenic resistance has occasionally been reported in specific biparental populations under controlled inoculation conditions [14], most evidence supports a polygenic model in which PM resistance is influenced by epistatic interactions, pathogen race specificity, and genotype-by-environment (G × E) effects [10,15]. Accordingly, the most informative PM resistance loci are those repeatedly detected across populations and environments, especially when supported by fine mapping or functionally plausible candidate genes.

Among the reported PM loci, chromosomes 1 and 5 emerge as the most important resistance regions. On chromosome 1, the major-effect QTL *Pm1.1* was fine-mapped to a 41.1 kb interval, within which two cysteine-rich receptor-like kinase (CRK) genes, *Csa1G029780* and *Csa1G029790*, were proposed as leading candidates based on their structural features and infection-responsive expression patterns [15]. This relatively narrow interval makes Pm1.1 one of the better-resolved PM loci and provides a useful foundation for further validation and marker development. On chromosome 5, the recessive locus Pm5.1 has been independently narrowed to an approximately 170 kb region containing *CsaMLO8* (*Csa010846*), a canonical susceptibility gene whose loss-of-function alleles confer broad-spectrum PM resistance [10,14]. Among the reported PM-associated genes, *CsaMLO8* is supported by the strongest functional evidence: CRISPR/Cas9 knockout enhances resistance in elite backgrounds [13,14], whereas transgenic complementation restores susceptibility [13]. Notably, this region also contains other defense-related genes, including *CsGy5G015660* and *CsPM5.2* (*Csa5G622830*), which have both been implicated in disease resistance [16,17]. Therefore, chromosome 5 should be viewed not as a single-gene region, but as a functionally complex resistance interval that includes both susceptibility-related and defense-associated components, which likely contributes to its repeated detection across studies.

In addition to these major loci, several medium- and minor-effect QTLs, such as *Pm1.2*, *Pm6.1*, and *Pm7.1*, may contribute to resistance improvement when considered in combination with major-effect loci [10]. Collectively, these findings indicate that PM resistance is currently the most genetically advanced among the three foliar disease systems in cucumber. In particular, loci such as *Pm1.1* and *Pm5.1*/*CsaMLO8* provide the clearest bridge between mapping results and translational potential, although the level of validation still differs substantially among reported genes and loci [13,14] (Table 1).

#### 2.1.2. QTLs for Downy Mildew Resistance

Downy mildew (DM), caused by the obligate biotroph *P. cubensis*, is among the most economically destructive foliar diseases of cucumber because of its rapid epidemic development and severe yield losses [18]. The genetic basis of DM resistance is highly complex. Although some biparental populations show monogenic or epistatic segregation patterns [19,20], most natural and elite germplasm exhibits quantitatively inherited resistance that is strongly influenced by pathogen race structure and environmental factors such as leaf age, humidity, and temperature [18,19,20,21,22]. Accordingly, the most informative DM loci are not simply those reported once, but those showing relatively large effects, repeated detection, or at least partial stability across different genetic backgrounds and evaluation conditions.

To date, more than 80 QTLs associated with DM resistance have been reported across all seven cucumber chromosomes (Table 2), with chromosomes 1, 2, 4, and 5 repeatedly implicated across independent studies. Among these loci, the dm4.1 region on chromosome 4 is one of the most intensively studied. This region comprises multiple sub-QTLs, explains 8.0–50.7% of phenotypic variance, and contains *CsLRK10L2*, an LRR-RLK gene induced during early *P. cubensis* infection and associated with enhanced ROS production and callose deposition in overexpression assays [20,21,23]. Chromosome 5 represents another major DM-associated region, where *dm5.1*, *dm5.2*, and *dm5.3* have been repeatedly detected and together explain approximately 5–30% of the phenotypic variance [18,20,21,24]. Within this broader chromosome 5 interval, *CsSIB1*, a sigma factor-binding protein, has been proposed as a positive regulator of SA signaling [25], whereas *CsaDMR6-2*, a homolog of the *Arabidopsis* susceptibility gene *DMR6*, represents another important candidate associated with recessive resistance when mutated [26]. Importantly, this region also contains a dense cluster of NBS-LRR genes, with more than 15 predicted NLRs within a 300 kb segment, highlighting how biologically plausible intervals may still remain difficult to resolve because of tight linkage among multiple candidates [18].

Despite the large number of mapped loci, several factors still limit causal resolution, including substantial pathogen race diversity that reduces phenotyping consistency [23,27], low field heritability caused by environmental noise [20,22], the persistence of large genomic intervals in some cases (for example, *dm1.1*) [18], and epistatic interactions that may obscure individual locus effects [20]. These limitations indicate that future progress in DM resistance genetics will depend less on the continued accumulation of mapped loci alone and more on improved phenotyping standardization, higher-resolution populations, and integrative analyses combining QTL mapping, GWAS, and haplotype-based approaches to refine candidate intervals. Overall, compared with PM resistance, DM resistance in cucumber has a broader mapped landscape but a lower degree of causal resolution, meaning that many reported loci should still be regarded as statistically supported regions rather than fully validated functional targets.

#### 2.1.3. QTLs for Target Leaf Spot Resistance

Compared with PM and DM, the genetic dissection of target leaf spot (TLS) resistance in cucumber remains much less advanced, both in the number of available studies and in mechanistic depth; nevertheless, recent work has begun to provide several important entry points for further analysis. Early screening studies identified partial resistance in accessions such as ‘Taiko’ and ‘Soldier’ [28], indicating that TLS resistance could be genetically captured and potentially introgressed. A major advance came from the study of resistant line D31, in which TLS resistance was shown to be controlled by a single recessive locus, *cca-3*, fine-mapped to a 79 kb interval on chromosome 6 containing *Csa6M375730.1*, a CC-NB-ARC-type gene structurally related to known NLRs [29]. This remains one of the highest-resolution TLS resistance intervals reported so far and provides a useful genetic anchor for future validation and fine dissection [29].

More recently, a large-scale evaluation of diverse cucumber germplasm identified elite TLS-resistant accessions, including CG28 and CG70, that showed relatively stable resistance across seasons and locations [30]. The same study identified three major loci, *gTLS5.1*, *gTLS5.2*, and *gTLS7.1*, and proposed several high-priority candidate genes, including *CsaV3_5G010580*, encoding a TIR-NB-LRR protein, and *CsaV3_7G026180*, encoding a WRKY transcription factor [30]. Cross-disease comparisons also suggest that TLS resistance may partially overlap with broader defense regulation. For example, *CsERF004*, an AP2/ERF transcription factor, has been implicated in responses to both DM and TLS [31], and chromosome 6, which harbors cca-3, also contains loci associated with PM and bacterial angular leaf spot resistance [32]. However, these overlaps should be interpreted cautiously, as positional or regulatory convergence does not by itself demonstrate a shared causal mechanism.

Despite these advances, TLS resistance research is still constrained by several major limitations, including the lack of standardized and well-characterized inoculum systems, the limited availability of highly resistant and agronomically useful germplasm, and the increasing fungicide resistance reported in *C. cassiicola* field populations [29,33,34]. Together, these constraints mean that TLS should currently be viewed as an emerging research frontier in cucumber disease resistance: promising loci and candidate regulators have been identified, but substantial work is still required to standardize phenotyping, validate causal genes, and connect mapping signals with mechanistic evidence. For this reason, TLS currently contributes more to defining future research priorities than to providing fully resolved resistance targets.

#### 2.1.4. Comparative Genomic Analysis of QTL Hotspots Across Diseases

**Figure 1:**
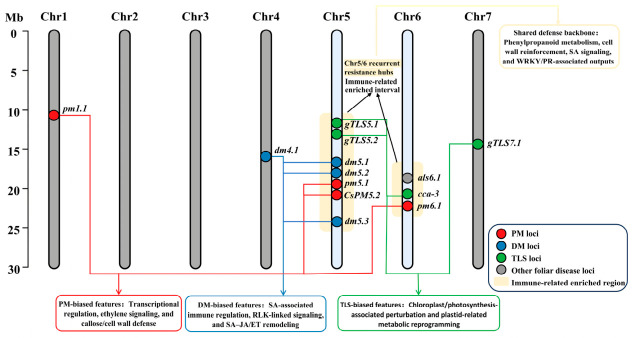
Integrated schematic of recurrent resistance hotspot regions and shared versus disease-biased defense modules across powdery mildew (PM), downy mildew (DM), and target leaf spot (TLS) in cucumber. Representative resistance-associated loci are mapped onto chromosomes Chr1–Chr7, highlighting the recurrent implication of Chr5 and Chr6 in multiple foliar disease-resistance studies. Shaded intervals indicate immune-related enriched regions corresponding to recurrent multi-disease hotspot signals. The lower panels summarize the disease-biased regulatory features inferred from comparative genetic and transcriptomic evidence, including PM-biased transcriptional regulation, ethylene signaling, and callose/cell wall defense; DM-biased SA-associated immune regulation, RLK-linked signaling, and SA–JA/ET remodeling; and TLS-biased chloroplast/photosynthesis-associated perturbation and plastid-related metabolic reprogramming. The shared defense backbone across PM, DM, and TLS is characterized by phenylpropanoid metabolism, cell wall reinforcement, SA signaling, and WRKY/PR-associated defense outputs. Importantly, positional overlap among loci does not necessarily imply identical causal genes or a single universal resistance mechanism; rather, it indicates that certain chromosomal regions repeatedly concentrate defense-associated components relevant to cross-disease resistance.

As in many plant genomes, resistance-associated loci in cucumber are not randomly distributed. In the context of foliar disease resistance, what is particularly notable is the repeated identification of major-effect regions on chromosomes 5 and 6 across independent studies and across multiple pathosystems (Figure 1; Table A1). Chromosome 5 contains overlapping loci associated with PM *(Pm5.1*, *Pm5.2*, *CsPM5.2*), DM (*dm5.1*, *dm5.2*, *dm5.3*), and TLS (*gTLS5.1*, *gTLS5.2*), making it the clearest example of a repeatedly implicated multi-disease resistance region in cucumber. This region is enriched in immune-related candidates, including NLR clusters, RLKs, and regulators linked to SA/JA signaling. Chromosome 6 also deserves attention because it contains *cca-3* for TLS, *Pm6.1* for PM, and *als6.1* for bacterial angular leaf spot resistance, further suggesting that this chromosome harbors an important concentration of defense-related loci [29,32].

However, positional overlap should not be interpreted as evidence of functional equivalence. For instance, *CsaMLO8* on chromosome 5 is specifically associated with PM susceptibility, whereas genes such as *CsSIB1* and *CsERF004* appear to participate in broader defense-related signaling through SA-or ET-associated pathways [25,31]. This coexistence of pathogen-specific and potentially broader defense-associated components within the same chromosomal neighborhoods highlights the need to move beyond positional overlap toward finer genetic and functional dissection. From a translational perspective, these repeatedly implicated regions should be regarded as priorities for comparative mapping, haplotype analysis, and future validation. Their value lies less in immediately defining breeding-ready targets than in helping narrow the search space for loci with potential relevance to multi-disease resistance. Thus, the comparative value of hotspot analysis lies not in suggesting that PM, DM, and TLS share a single resistance mechanism, but in showing that certain chromosomal regions repeatedly emerge as focal points for further cross-disease investigation.

### 2.2. Complementary Evidence from GWAS

Genome-wide association studies (GWAS) complement biparental QTL mapping by capturing allelic variation from diverse germplasm and, in principle, offering higher mapping resolution. In cucumber disease-resistance research, however, the interpretability of GWAS signals depends strongly on population structure, marker density, phenotyping quality, and the ability to control environmental and pathogen-related variation [35,36,37,38]. Therefore, the main value of GWAS in this field lies not simply in reporting additional associated loci, but in helping prioritize genomic regions that are repeatedly detected, biologically plausible, or supported by independent QTL evidence.

#### 2.2.1. Methodological Considerations

In practice, disease-resistance GWAS in cucumber is especially sensitive to four methodological factors: the composition of the association panel, marker density, phenotyping consistency, and the statistical control of population structure and relatedness [35,36,37,38]. Because disease responses are often influenced by race specificity and environmental fluctuation, inconsistent phenotyping can substantially weaken signal stability. As a result, GWAS findings are most convincing when they are reproducible across populations or converge with independent QTL and candidate-gene evidence.

#### 2.2.2. GWAS Signals for PM, DM, and TLS Resistance

GWAS signals reported for cucumber foliar disease resistance differ substantially in their degree of resolution and interpretability across pathosystems. For powdery mildew (PM), GWAS evidence is currently the most informative when it converges with previously mapped major-effect regions, especially on chromosome 5, where association signals reinforce the importance of loci linked to *CsaMLO8*, *CsGy5G015660*, and related defense-associated genes [10,13,14,16,17]. In this context, GWAS contributes less by revealing entirely new architectures than by increasing confidence in already prioritized regions and helping narrow the list of biologically plausible candidates.

For downy mildew (DM), GWAS has broadened the landscape of associated loci, particularly on chromosomes 4 and 5, and has helped highlight intervals that overlap with repeatedly detected QTL regions [18,20,21,24,25,26]. However, the biological interpretation of many DM-associated signals remains less straightforward than in PM, because the phenotype is highly sensitive to pathogen race structure, environmental conditions, and the density of defense-related gene clusters within broad intervals. As a result, GWAS in DM is most valuable when treated as a layer of supportive evidence for prioritizing recurrent regions rather than as stand-alone proof of causal loci.

For target leaf spot (TLS), GWAS is even more clearly exploratory in value. Recent association analyses have identified several TLS-associated loci and candidate genes [30]. These results are important because they substantially expand the still-limited genetic framework available for TLS resistance in cucumber. Nevertheless, most TLS-associated signals should presently be regarded as promising leads rather than functionally resolved determinants, especially in the absence of equivalent levels of validation comparable to those available for the best-characterized PM loci.

Taken together, GWAS contributes most effectively to cucumber foliar disease-resistance research when its signals are interpreted comparatively rather than cumulatively. In other words, the key question is not how many additional loci are detected, but which association signals repeatedly coincide with independent QTL evidence, biologically plausible candidate genes, or functionally informative genomic regions (Table A2).

#### 2.2.3. Integrating QTL and GWAS Evidence

QTL mapping and GWAS should be regarded as complementary rather than interchangeable approaches for dissecting cucumber foliar disease resistance. QTL mapping is particularly useful for detecting genomic regions with measurable effects in controlled biparental populations, whereas GWAS can capture allelic diversity from broader germplasm panels and, in some cases, provide finer positional resolution [35,36,37,38]. However, neither approach alone is sufficient to establish causal inference. The most convincing evidence emerges when QTL intervals and GWAS signals converge within the same genomic region and are further supported by biologically plausible candidate genes or functional data.

Under this framework, powdery mildew (PM) currently provides the clearest example of successful evidence integration in cucumber. In particular, the repeated detection of chromosome 5 loci across independent mapping studies, together with GWAS support and functional evidence for genes such as *CsaMLO8*, has created a relatively coherent genetic picture compared with the other foliar disease systems [10,13,14,16,17]. This does not mean that all PM-associated loci are equally resolved, but it does indicate that several regions have moved beyond purely statistical association toward stronger genetic and biological support.

For downy mildew (DM), integrative evidence is informative but less conclusive. Multiple QTLs and GWAS signals repeatedly implicate chromosomes 4 and 5, suggesting that these regions are important components of the DM resistance architecture [18,20,21,24,25,26]. Nevertheless, many of these intervals remain broad, gene-dense, and highly sensitive to race-specific and environmental effects, which limits the extent to which positional overlap alone can be interpreted as mechanistic confirmation. Thus, for DM, integration mainly improves regional prioritization rather than establishing fully resolved causal targets.

For target leaf spot (TLS), the integrative framework is even more preliminary. QTL mapping has identified cca-3 as an important high-resolution locus, while recent GWAS has expanded the number of candidate regions and genes, including *gTLS5.1*, *gTLS5.2*, and *gTLS7.1* [29,30]. At present, however, the principal contribution of combining these datasets is to define promising research directions rather than to confirm a stable set of validated resistance determinants.

Overall, the value of integrating QTL and GWAS evidence lies not in maximizing the number of reported loci, but in stratifying them by confidence. Genomic regions supported by repeated detection, cross-method convergence, and biologically plausible candidates should be prioritized for fine mapping and validation, whereas loci supported by only one analytical framework should be interpreted more cautiously. In this sense, integrative analysis provides a practical way to distinguish comparatively robust resistance regions from those that remain provisional.

Overall, current genetic evidence indicates that cucumber foliar disease resistance is characterized by substantial heterogeneity in both mapping depth and biological resolution. Among the three major pathosystems discussed here, PM resistance currently shows the clearest convergence of QTL, GWAS, and functional evidence, particularly around chromosome 5. By contrast, DM resistance has a broader but less resolved genetic landscape, with many loci still requiring finer validation despite repeated detection across studies. TLS resistance remains the least mature of the three systems, but recent mapping and association studies have begun to define a set of promising entry points for future research. Taken together, these findings suggest that the main challenge is no longer the mere accumulation of additional loci, but the prioritization of those genomic regions that repeatedly emerge across methods and studies and can be advanced toward causal validation.

## 3. Candidate Genes and Molecular Defense Modules Underlying Cucumber Foliar Disease Resistance

### 3.1. From Mapped Loci to Functional Interpretation

The genetic evidence summarized in Chapter 2 helps identify which chromosomal regions and candidate loci deserve closer functional attention. However, mapped loci alone do not explain how cucumber responds to diverse foliar pathogens at the molecular level. To move from positional evidence to biological interpretation, it is necessary to examine the major classes of resistance-related genes and regulatory modules that repeatedly appear near prioritized loci or in disease-responsive transcriptomic studies. In cucumber, the most relevant components include nucleotide-binding leucine-rich repeat receptors (NLRs), receptor-like kinases (RLKs), transcription factors (TFs), and pathogenesis-related (PR) proteins. Together, these elements contribute to pathogen recognition, signal transmission, transcriptional reprogramming, and downstream defense execution, although the strength of evidence varies considerably among genes, families, and pathosystems.

### 3.2. Major Resistance-Related Gene Classes and Defense Modules

#### 3.2.1. NLR- and Receptor-Associated Components

Among the resistance-related gene classes identified in cucumber, NLRs remain important candidates because of their established roles in intracellular pathogen recognition and effector-triggered immunity. However, the cucumber NLR repertoire appears relatively compact compared with that of several other model and crop species, and current evidence does not support treating all NLR-rich intervals as functionally equivalent resistance hotspots [39,40]. Instead, the most useful interpretation is that some prioritized genomic regions—especially on chromosome 5—contain clusters of immune-related genes that warrant finer dissection, particularly where they overlap with repeatedly mapped disease-resistance loci [18,39,40].

This point is especially relevant in the chromosome 5 region associated with PM and DM resistance, where NLR-rich intervals occur near repeatedly detected loci such as *dm5.2* and *dm5.3* [18,40]. Such co-localization is informative, but it should be interpreted cautiously: the physical proximity of NLRs to mapped QTLs does not by itself establish causality, nor does it demonstrate that neighboring resistance- and susceptibility-related genes operate through the same mechanism. Rather, these regions should be viewed as promising targets for future functional validation, haplotype dissection, and candidate prioritization.

RLKs provide an additional and often more immediately interpretable layer of defense-related candidates. In cucumber, LRR-RLKs constitute a large and functionally diverse receptor class involved in the perception of extracellular pathogen-associated molecular patterns and the activation of pattern-triggered immunity [41,42]. Orthologs of well-characterized receptors such as *CsFLS2*, *CsCERK1*, and *CsLYK5* have been reported to respond to infection by PM, DM, and TLS pathogens, with stronger or more sustained induction in resistant genotypes in some studies [41]. These observations suggest that RLK-associated signaling may help explain part of the variation captured by mapped resistance loci, particularly in cases where QTL regions contain receptor-like candidates such as *CsLRK10L2* [20,23,41,42]. Even so, most RLK-linked interpretations in cucumber should still be regarded as mechanistic hypotheses supported by expression and positional evidence rather than fully resolved functional models (Table A3).

#### 3.2.2. Transcriptional Regulators and Signaling Integration

Beyond receptor-associated components, transcription factors represent a major regulatory layer linking pathogen perception to downstream defense outputs. In cucumber, members of transcription factor families such as WRKY, NAC, and AP2/ERF are repeatedly identified in disease resistance-related expression studies and candidate gene analyses [43,44]. Their importance lies not in functioning as isolated markers of resistance, but in integrating hormone signaling, defense gene activation, and stress-responsive transcriptional programs.

Among these families, WRKY factors are particularly prominent. Genes such as *CsWRKY40*, *CsWRKY50*, and *CsWRKY70* have been implicated in PM and DM responsive expression networks and are generally associated with salicylic acid-related defense regulation [44]. Likewise, *CsERF004* provides a useful example of how AP2/ERF family regulators may connect disease-specific and broader defense-associated responses, as it has been discussed in relation to both DM and TLS resistance [31,43]. Other transcription factor families likely contribute more specialized functions: NAC factors have been linked to hypersensitive response and structural defense reinforcement, bZIP proteins to the integration of light-related and systemic signaling, and MYB factors to phenylpropanoid-associated defenses such as lignin and flavonoid accumulation [43,44].

Although these observations support the view that transcription factors are key components of cucumber disease resistance, the current evidence remains uneven in depth and validation. For many genes, expression responsiveness and network position are clearer than direct causal function. Accordingly, transcription factor families are best treated as candidate regulatory modules that help bridge mapped loci and defense phenotypes, rather than as uniformly validated determinants of resistance.

#### 3.2.3. Downstream Defense Effectors and Pathogenesis-Related Proteins

Pathogenesis-related proteins represent a downstream defense layer that is more closely associated with defense execution than with resistance specificity itself. Cucumber contains orthologs of multiple PR families, and several of them—particularly PR1, PR2, and PR5—are repeatedly reported as induced during PM, DM, and TLS infection [43,45,46]. This recurrent induction suggests that they contribute to a broadly shared defense response, even though their expression alone should not be taken as proof of resistance efficacy.

Other PR-associated components may display more context-dependent behavior. For example, some PR10 isoforms appear to be more strongly associated with PM-responsive expression, whereas PR14-type lipid transfer proteins have been discussed in relation to TLS-associated responses [43,45]. Chitinase-related PR families (PR3, PR4, PR8, and PR11) are also broadly relevant, and the reported effect of Chi2 overexpression on resistance to both PM and Fusarium wilt provides one of the clearer examples of a multifunctional downstream defense component in cucumber [47].

Taken together, these observations indicate that PR proteins should be viewed primarily as markers and mediators of activated defense states rather than as stand-alone explanations for disease-resistance loci. Their value in this review lies in showing how prioritized genomic regions and upstream regulatory candidates may ultimately connect to measurable antimicrobial, structural, or stress-mitigation responses.

#### 3.2.4. Interim Synthesis of Major Defense-Related Gene Classes

Overall, current evidence supports a layered view of cucumber foliar disease resistance in which receptor-associated genes, transcriptional regulators, and downstream defense effectors contribute to partially connected but unevenly resolved defense modules. Some genes, such as *CsaMLO8*, *CsLRK10L2*, *CsERF004*, and *Chi2*, already provide useful anchors for linking genetic signals to biological function, whereas many other candidates remain supported primarily by positional overlap, expression responsiveness, or family-level plausibility. This distinction is important: the field is now moving beyond the simple cataloging of resistance-related genes and toward the more demanding task of identifying which candidates have sufficient multi-layer evidence to support functional validation and eventual translational use (Table A4).

### 3.3. Shared Defense Signaling Pathways and Cross-Disease Regulatory Convergence

Although cucumber foliar diseases differ in pathogen lifestyle, infection strategy, and host interaction dynamics, current evidence suggests that at least part of their resistance architecture converges on a limited number of shared defense-associated pathways. These include salicylic acid (SA) related signaling, jasmonic acid/ethylene (JA/ET) associated responses, reactive oxygen species (ROS) accumulation, callose deposition, phenylpropanoid metabolism, and transcriptional reprogramming mediated by defense-responsive transcription factors [25,31,41,42,43,44,45,46]. The extent to which each pathway contributes varies among pathosystems, but together they provide a functional framework for interpreting why some genomic regions repeatedly emerge in comparative mapping analyses.

Among these pathways, SA-associated signaling appears particularly relevant to resistance against PM and DM, where several candidate regulators and responsive genes have been linked to defense activation. For example, *CsSIB1* has been discussed as a positive regulator of SA signaling in the chromosome 5 DM associated region [25], while multiple WRKY factors implicated in PM and DM responsive expression networks are also commonly interpreted in relation to SA-dependent defense regulation [44]. However, such associations should not be overstated: although these genes are biologically plausible components of SA-linked defense, the strength of direct functional validation is not uniform across all candidates.

JA and ET-related pathways may contribute more variably, particularly in the context of tissue damage, necrotrophic challenge, or broader stress integration. This is relevant to TLS, where regulators such as *CsERF004* have been proposed as part of cross-disease defense signaling and may help connect TLS-associated responses with more general immune regulatory networks [31,43]. Even so, current evidence is still insufficient to conclude that PM, DM, and TLS share a single regulatory architecture. A more cautious interpretation is that these diseases partially recruit overlapping signaling components while retaining important pathogen-specific differences in timing, intensity, and downstream output.

Additional defense-associated processes, including ROS burst, cell wall reinforcement, and phenylpropanoid accumulation, further illustrate this partial convergence. For instance, the proposed role of *CsLRK10L2* in promoting ROS production and callose deposition during DM-related responses suggests how receptor-associated candidates can be linked to measurable cellular defense outputs [20,21,23]. Similarly, MYB and NAC-associated regulation of lignification and structural defense provides a plausible bridge between upstream signaling and the physical restriction of pathogen spread [43,44]. These downstream processes are relevant across multiple foliar diseases, but their contribution is likely shaped by pathogen biology and the specific host background in which resistance is evaluated.

**Figure 2:**
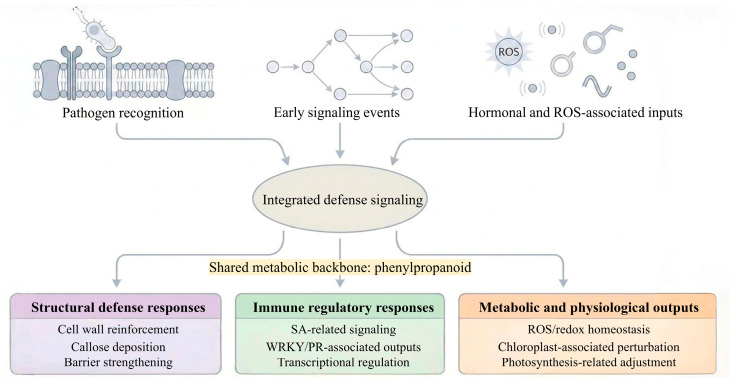
Conceptual framework of shared defense signaling and downstream response modules in cucumber foliar disease resistance. The schematic summarizes a mechanistic model for cucumber responses to major foliar diseases, integrating pathogen recognition, early signaling events, and hormonal/ROS-associated inputs into a shared defense signaling network. Downstream responses are organized into three partially overlapping modules, including structural defense responses (cell wall reinforcement, callose deposition, and barrier strengthening), immune regulatory responses (SA-related signaling, WRKY/PR-associated outputs, and transcriptional regulation), and metabolic/physiological outputs (ROS/redox homeostasis and chloroplast-associated perturbation). Phenylpropanoid metabolism is highlighted as a shared component of the defense backbone. Importantly, these pathways should be interpreted as partially shared but differentially weighted across pathosystems, rather than as evidence of a single universal resistance mechanism.

Taken together, the available evidence supports a model in which cucumber foliar disease resistance is built from partially shared but differentially weighted defense modules. In this view, repeated QTL or GWAS detection in the same chromosomal region may reflect the local concentration of genes connected to broadly important defense pathways, rather than a single universal resistance mechanism. This distinction is essential for interpreting cross-disease overlap correctly and for identifying which candidate genes are most suitable for targeted validation (Figure 2).

### 3.4. Cross-Disease Re-Analysis of Public Transcriptomic Data

Current studies on powdery mildew, downy mildew, and target leaf spot in cucumber have largely remained within single-disease frameworks, primarily revealing the response characteristics specific to each disease, while lacking direct cross-disease comparisons under a unified analytical framework [25,48,49]. Therefore, in order to identify defense processes potentially shared across multiple diseases, compare disease-specific response emphases, and further prioritize candidate nodes and defense markers worthy of attention, we re-analyzed three public cucumber RNA-seq datasets from NCBI under a unified pipeline in R: GSE81234 (PM, 48 h) [48], SRP383809 (DM, 72 h) [25], and SRP117262 (TLS, 24 h) [49]. The results showed that all three diseases induced pronounced defense-related transcriptional reprogramming and, on the basis of partially shared defense processes, also exhibited distinct downstream response emphases. These findings not only revealed a common defensive foundation across the three diseases but also further clarified the major directions of their response divergence, thereby providing a basis for subsequent candidate gene screening and studies on multi-disease resistance mechanisms.

First, a total of 218 cross-disease shared differentially expressed genes (DEGs) were identified across the three datasets (*p* < 0.001, hypergeometric test), indicating that cucumber activates a relatively stable shared defensive transcriptional response following infection by powdery mildew, downy mildew, and target leaf spot. Functional enrichment analysis of these shared DEGs showed that they were mainly involved in phenylpropanoid-related metabolism (GO:0009698), salicylic acid-related signaling (GO:0009751), and ammonia-lyase activity (GO:0016841), suggesting the existence of a cross-disease recognizable common defense core among the three foliar diseases [50,51,52]. This core mainly points to phenylpropanoid-related metabolism, salicylic acid-related responses, and structural defense reinforcement, indicating that although the three diseases differ in pathogenic mode and host response dynamics, cucumber may still preferentially mobilize a relatively conserved set of basal defense processes under foliar disease stress [50,51,52]. This shared defensive background also provides an essential reference for subsequent comparisons of divergence in downstream pathway configuration among different diseases.

Analysis of disease-specific pathways further showed that, beyond the shared defense core, the three diseases displayed different functional emphases in their downstream responses. PM was specifically enriched in sequence-specific DNA binding (GO:0043565) and ethylene signaling (GO:0009873), suggesting that its response is more closely associated with transcriptional regulation, ethylene-related defense, and callose/cell wall-related processes [17]. In contrast, DM was enriched in defense response to bacterium (GO:0042742) and heme binding (GO:0020037); together with the reported framework of CsSIB1-dm5.3-mediated SA enhancement and relative suppression of JA/ET, this result is consistent with the more prominent immune-regulatory features observed in DM [25,53]. TLS, by contrast, showed more pronounced enrichment in plastid- and photosynthesis-related pathways (GO:0009536, GO:0015979), indicating that, in addition to general defense activation, its transcriptional response more prominently involves chloroplast/photosynthesis-related perturbation and broader metabolic remodeling [49,54]. These differences indicate that cucumber resistance responses to different foliar diseases are not merely simple repetitions of a shared defense program, but rather exhibit distinct response emphases against a common defensive background.

On the basis of the above pathway comparison, candidate nodes and defense markers were further screened. The STRING protein–protein interaction network constructed from the 218 shared DEGs (confidence > 0.7) showed that *WRKY2*, *WRKY23*, and *Csa_3G716870* had relatively high connectivity and may therefore serve as candidate hub nodes worthy of priority attention within the shared defensive background [44,55]. Further PR gene heatmap analysis indicated that PR1 and PR5 may serve as common defense markers across PM, DM, and TLS, whereas PR10 was more strongly associated with PM, consistent with its potential role in responses to biotrophic colonization (Table A5) [43,45]. These results indicate that cross-disease comparative analysis not only helps identify the common defense core and disease-response divergence, but also advances the comparative results further to the level of candidate nodes, molecular markers, and research prioritization.

Compared with the original single-disease studies, this cross-disease re-analysis extracted several key aspects that are not easily discernible within a single-disease framework [17,25,48,49,54]. Specifically, the 218 shared differentially expressed genes and their common enrichment patterns outlined a potentially shared defense-related core among these three foliar diseases [50,51,52]; the relative emphases of enriched pathways in different diseases further revealed divergence in downstream response regulation among powdery mildew, downy mildew, and target leaf spot [17,25,49,53,54]; and the expression patterns of hub genes such as *WRKY2*, *WRKY23*, and *Csa_3G716870* in the STRING protein–protein interaction network, together with the comparative results for PR1, PR5, and PR10, provided more explicit candidate targets for subsequent functional validation [43,44,45,55]. Unlike the original studies, which mainly described the transcriptomic features of each disease separately, the present study placed powdery mildew, downy mildew, and target leaf spot within a unified analytical framework for comparison, thereby yielding several integrative conclusions that are not readily apparent from any single study alone. These include the inference of a cross-disease shared defense foundation centered on salicylic acid-related signaling and phenylpropanoid-related metabolism [50,51,52], the comparative delineation of distinct downstream response priorities for powdery mildew, downy mildew, and target leaf spot [17,25,49,53,54], and the further identification of shared hub candidate genes and broad-spectrum defense markers that merit priority validation [43,44,45,55].

**Figure 3:**
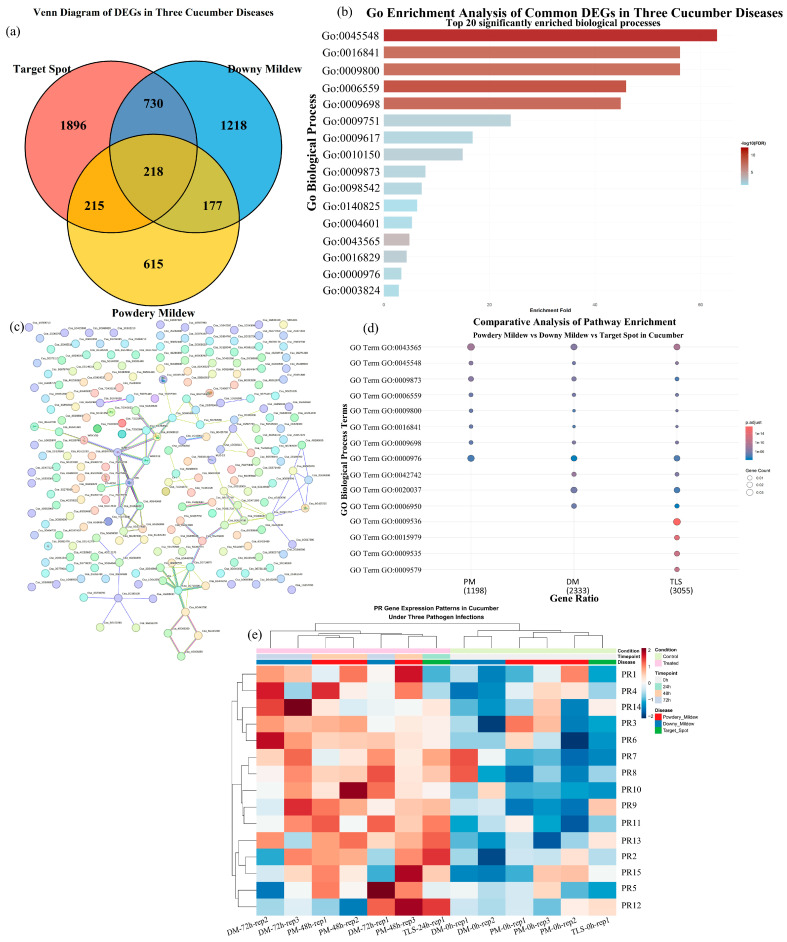
Cross-disease transcriptomic integration identifies a shared defense core and disease-biased response features in cucumber foliar diseases. Comparative re-analysis of PM, DM, and TLS transcriptomes revealed a set of shared differentially expressed genes associated with phenylpropanoid metabolism, salicylic acid-related defense, and ammonia-lyase activity, supporting the presence of a common defense-associated backbone. At the same time, the three pathosystems differed in the relative emphasis of downstream responses, with PM showing stronger transcriptional and ethylene-related features, DM showing a more pronounced immune-regulatory signature, and TLS showing greater association with plastid-/photosynthesis-related perturbation and metabolic remodeling. Network and PR-gene analyses further highlighted candidate shared hub regulators and both common and disease-biased defense markers. (**a**) DEG overlap among PM, DM, and TLS: Venn diagram showing the number of differentially expressed genes (DEGs) common to the three diseases. Red, blue, and yellow circles represent Target Spot (DM), Downy Mildew (PM), and Powdery Mildew (TLS) diseases, respectively. The overlap shows the shared DEGs among all diseases. (**b**) GO enrichment of shared DEGs: Top 20 significantly enriched biological processes of shared DEGs, with color intensity representing the enrichment level (from light blue for low to dark red for high enrichment). (**c**) PPI network of shared DEGs: Protein-protein interaction network depicting shared DEGs, with nodes color-coded based on biological process association, showing the interactions between proteins in these processes. (**d**) Comparative enrichment of disease-biased GO terms: Comparative analysis of the enrichment of disease-biased GO terms. The size of the dots represents the gene count, and the color reflects the enrichment factor, with blue indicating low enrichment and red indicating high enrichment. (**e**) PR gene expression patterns across the three diseases: Heatmap showing the expression of PR genes across the three diseases. Red indicates high expression, and blue indicates low expression, with rows representing different PR genes and columns representing various experimental conditions (DM, PM, TLS).

Among these findings, the comparative positioning of target leaf spot is particularly noteworthy. Although previous TLS studies have reported that its response involves plastid-related processes and photosynthetic perturbation within a single-disease framework [49,54], the present cross-disease comparison further led to a new integrative conclusion: compared with powdery mildew and downy mildew, target leaf spot exhibits a more pronounced transcriptomic bias toward plastid-related processes, photosynthetic perturbation, and metabolic reprogramming. This suggests that plastid/photosynthesis-related changes are not merely general accompanying responses in TLS, but rather represent one of the characteristic molecular features distinguishing it from the other two foliar diseases. Because this conclusion could not be directly derived from the original single-disease studies, it constitutes an important new insight from the present work regarding the response characteristics of TLS, and provides a clearer entry point for subsequent candidate gene screening and functional validation aimed at elucidating the resistance mechanisms of target leaf spot [49,54]. However, current evidence related to target leaf spot still mainly derives from comparative transcriptomic analyses, and functional validation of the relevant candidate genes and pathways remains limited. Nevertheless, the present study has further highlighted the relative importance of plastid-related processes, photosynthetic perturbation, and metabolic reprogramming in the response to target leaf spot [49,54], thereby providing a more focused basis for subsequent candidate gene screening and functional studies (Figure 3a–e, Table A6).

### 3.5. Toward Predictive, Breeding-Ready Networks

Current multi-omics integration research remains largely at a descriptive stage. To advance predictive breeding, we propose a shift from “association maps” to “causal blueprints.”

First, priority should be given to key hub genes supported by cross-validation across multiple layers of evidence. Genes that are consistently identified in two or more omics layers should be regarded as priority targets for breeding. For example, if *CsWRKY50* is simultaneously detected as a differentially expressed gene (DEG), associated with a ChIP-seq binding peak, involved in protein–protein interactions, and significantly correlated with metabolite changes, it can be considered a high-confidence core candidate gene with strong application potential.

Second, a functional allelic series should be established for these key hub genes. For each core gene, natural and edited alleles should be systematically characterized in terms of gradients in expression level, functional activity, and resistance phenotype. This would make it possible to define the relationship among allelic variation, functional strength, and disease-resistance performance, thereby providing a more precise basis for genomic prediction of resistance intensity.

Third, environmental factors should be incorporated into the multi-omics network analysis framework. By integrating genotype-by-environment (G × E) interaction data, such as temperature-dependent expression changes in CsMLO1, it would be possible to construct climate-resilient resistance network models with both environmental responsiveness and stable predictive power.

Overall, this framework would transform multi-omics from a discovery tool into a breeding-oriented design platform, thereby providing more direct and robust theoretical support for molecular marker development, gene-editing target selection, and resistance gene pyramiding strategies.

## 4. From Gene Discovery to Breeding Deployment: Bridging the Translational Divide

The ultimate objective of cucumber disease resistance functional genomics is not merely gene discovery, but the robust, scalable deployment of validated resistance mechanisms into elite breeding pipelines—yielding varieties with durable, field-ready resistance that maintains high yield, quality, and adaptability. While a mature technical ecosystem now exists for gene function validation, marker-assisted selection (MAS), and precision genome editing, the translational pipeline from “candidate locus” to “commercial variety” remains fragmented, inefficient, and bottlenecked by systemic gaps—commonly termed the “valley of death” This gap reflects not only technical hurdles but also misalignments in scale, standards, and incentives between basic research, pre-breeding, and commercial breeding.

### 4.1. Functional Validation: Rigor, Relevance, and Readiness

Functional validation serves as the critical gatekeeper between genomic discovery and breeding utility. Its purpose is not simply to confirm causality in controlled settings, but to assess whether a candidate gene or allele delivers breeding-relevant outcomes: broad-spectrum efficacy across pathogen races, stability across environments and genetic backgrounds, and neutrality—or enhancement—of key agronomic traits. Current strategies include transgenic overexpression, CRISPR/Cas9-mediated knockout or knock-in, RNA interference (RNAi), virus-induced gene silencing (VIGS), and Agrobacterium-mediated transient expression—each selected based on biological question, speed requirement, regulatory context, and trait architecture (Table A7).

For positive regulators, overexpression of *CYP82D47* and *CsCSE1* confers enhanced resistance to powdery mildew (PM), *Fusarium* wilt, and *P. xanthii* infection, accompanied by coordinated upregulation of PR proteins and SA/ethylene-responsive defense genes [56,57]. Heterologous overexpression of *RCC2* and *CsCBS* similarly enhances resistance to gray mold, downy mildew (DM), and *Cercospora* leaf spot [58,59]. In contrast, targeted disruption of susceptibility (*S*) genes via CRISPR/Cas9 has yielded the most translationally advanced outcomes: eIF4E loss-of-function alleles confer near-immunity to multiple potyviruses and are already deployed in commercial cucumber hybrids [60,61,62]; *CsaMLO8* knockout lines exhibit stable, broad-spectrum PM resistance across diverse physiological races and under semi-commercial greenhouse and field conditions [63,64]. Rapid, transient tools—including VIGS, amiRNA, and transient overexpression—enable high-throughput screening of candidates prior to stable transformation, having successfully elucidated roles for *CsIVP-CsNIMIN1*, *CsCSE1*, *CsMLO1/2*, *CmLOX10*, and *CmPDS*, and revealed miRNA–target modules (*miR164d*, *miR396b*) governing lignin biosynthesis and resistance [57,65,66,67,68,69].

Crucially, rigorous functional validation must be designed for translational readiness:

Broad-spectrum testing: Evaluation against ≥ 3 defined pathogen races under standardized inoculation protocols—not just one lab isolate;

Environmental robustness: Multi-location, multi-year field trials—not just growth chamber assays—to capture genotype-by-environment (G × E) interactions;

Agronomic integration: Concurrent monitoring of yield components (fruit number/weight), quality traits (firmness, sugar content, shelf life), and stress resilience (drought, heat)—to detect linkage drag or pleiotropic trade-offs early.

*CsaMLO8* stands as the paradigmatic success story. Fine mapping of the *Pm*5.1 QTL converged on *CsaMLO8* as the causal *S* gene [63,64,70]. CRISPR knockout conferred strong, race-nonspecific PM resistance, with histological evidence of enhanced papillae formation, callose deposition, and reduced hyphal penetration [63,64]. Phylogenetic analysis confirmed its functional orthology to *AtMLO2/6/12*, and natural loss-of-function alleles were identified in resistant germplasm [64]. Critically, the KASP marker *MLO8-KASP1* enabled efficient marker-assisted backcrossing into elite backgrounds; resulting lines maintained >98% recurrent parent genome recovery and delivered stable, high-level PM resistance across 3 years and 5 locations—with no significant penalty on yield or fruit quality [63,64]. This represents a fully validated, end-to-end translational pathway: QTL—gene—mechanism—marker—elite line—field performance.

### 4.2. Marker-Assisted Selection (MAS): Precision, Scalability, and Pragmatic Integration

MAS is now an indispensable, high-precision tool in cucumber breeding—but its power lies not in replacing phenotyping, but in *strategically augmenting* it. Modern MAS leverages diverse marker systems—KASP (for single-SNP, high-throughput genotyping), SSR (for pedigree tracking and diversity analysis), SNP chips (for background selection and QTL fine-mapping), and CAPS/InDel (for cost-effective co-dominant assays)—tailored to specific breeding objectives [63,64,71].

The *MLO8-KASP1* assay exemplifies best practice: it enables rapid, low-cost, high-fidelity identification of the *mlo*8 allele in BC_1_F_2_ populations, while SNP chip-based background selection ensures >98% genome recovery of the elite recurrent parent within 3–4 generations [63]. For multi-disease resistance, MAS enables precise pyramiding: simultaneous introgression of *Pm*5.1 (PM) and *dm*5.1 (DM) resistance alleles using tightly linked markers has been successfully implemented in commercial programs, yielding lines with dual resistance without compromising horticultural performance [71].

However, MAS is not infallible. Linkage between marker and causal variant can break due to recombination—especially in large QTL intervals or divergent genetic backgrounds—and QTL effects are inherently environment-sensitive. Therefore, MAS must be embedded in a *tiered selection strategy*:

Early-generation pre-screening using MAS to enrich for target alleles;

Mid-generation phenotypic screening under controlled, race-defined inoculation to confirm functional efficacy;

Late-generation multi-environment field evaluation to validate stability, yield, and quality.

This integrated approach maximizes efficiency while safeguarding against false positives and environmental instability (Table 3).

### 4.3. Genome Editing: From Targeted Mutation to Modular Trait Engineering

CRISPR/Cas9 editing has transitioned from a proof-of-concept tool to a core breeding technology in cucumber. Its greatest impact lies in *S*-gene editing: *CsaMLO8* and eIF4E edits deliver broad-spectrum, durable resistance without transgenes, and crucially, without yield penalties—fulfilling the “non-GMO” regulatory pathway in many jurisdictions [60,61,62,63,64]. Looking ahead, next-generation editing expands the scope: multiplex editing enables simultaneous modification of multiple *S* genes (such as *CsMLO1/2/8*) or stacking of resistance modules; base editing allows precise amino acid substitutions to tune gene function rather than abolish it; and DNA-free editing (ribonucleoprotein delivery) eliminates regulatory concerns associated with foreign DNA integration [72,73].

Yet practical deployment faces persistent challenges: off-target edits remain a concern despite the improved fidelity of Cas variants; transformation and regeneration efficiencies are still low (<15%) in many elite lines; functional redundancy among paralogs can mask phenotypic effects; and unintended pleiotropy—such as altered flowering time or fruit development—requires careful assessment. For understudied diseases like target leaf spot (TLS), a pragmatic, stepwise workflow is recommended: (i) transcriptome-guided candidate prioritization; (ii) rapid VIGS/RNAi screening in elite germplasm to triage candidates; (iii) stable CRISPR editing only for top-tier, high-confidence targets; (iv) rigorous multi-environment field evaluation. This conserves resources and focuses editing efforts where they deliver maximum return.

### 4.4. The Valley of Death: Systemic Bottlenecks and Integrated Solutions

The translational failure rate remains stark: high-throughput studies routinely identify dozens to hundreds of candidate resistance loci, yet <1% progress to commercial deployment. The full cycle—from QTL discovery to registered variety—typically spans 8–12 years and incurs high attrition: candidates fail at functional validation (instability, pleiotropy), field testing (G × E breakdown), or regulatory/commercial adoption (cost, scalability, market fit) [71,72]. This “valley of death” is not merely technical—it is systemic, rooted in:

Disciplinary silos: Basic researchers optimize for statistical significance and mechanistic insight; breeders prioritize heritability, stability, and integration into existing pipelines;

Resource misalignment: Academic grants favor discovery over translation; industry lacks access to cutting-edge tools or germplasm;

Regulatory fragmentation: Divergent global frameworks for edited crops hinder international deployment;

Infrastructure gaps: Lack of standardized, high-throughput phenotyping platforms for complex traits like TLS resistance.

**Figure 4:**
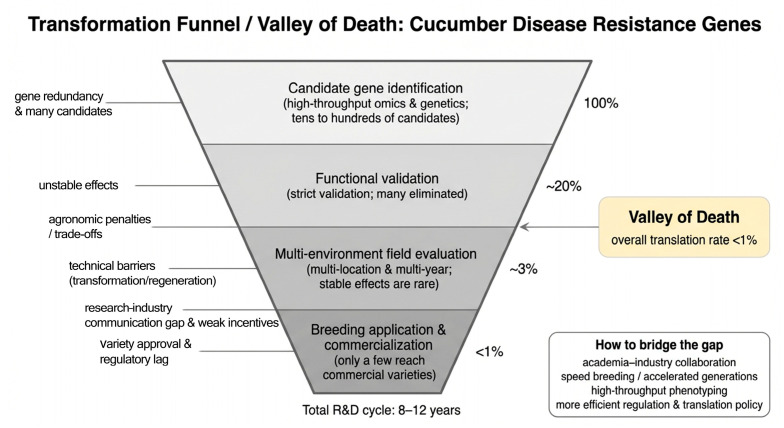
Funnel diagram illustrating the translational bottleneck (“Valley of Death”) in transferring cucumber disease-resistance genes from discovery to breeding application. High-throughput omics and genetic studies typically generate tens to hundreds of candidate genes, but only a subset proceeds to strict functional validation, and an even smaller fraction shows stable performance in multi-location and multi-year field evaluation. Ultimately, <1% of initial candidates are incorporated into commercial cultivars, and the overall R&D cycle commonly spans 8–12 years. This steep attrition reflects biological and technical constraints, including gene redundancy, unstable effects, and agronomic trade-offs (including pleiotropic penalties), as well as technical barriers in transformation/regeneration systems, research–industry communication gaps, weak incentives, and delays in variety approval and regulatory processes.

Bridging this divide demands integrated, mission-driven innovation:

Establish pre-breeding hubs that co-locate genomicists, pathologists, and breeders to jointly design validation pipelines aligned with breeding goals;

Deploy accelerated breeding (speed breeding, doubled haploids) and AI-powered high-throughput phenotyping (hyperspectral imaging, automated disease scoring) to compress generation cycles and improve data quality;

Advocate for science-based, product-focused regulatory pathways for SDN-1/SDN-2 edited crops;

Build shared, open-access databases linking QTLs, edited alleles, field performance data, and regulatory status—creating a transparent, community-wide knowledge infrastructure (Figure 4).

## 5. Extending Insights Across the Cucurbitaceae

While cucumber serves as the foundational model, its insights must be translated across the family. This section synthesizes disease resistance advances in watermelon, melon, and pumpkin and, critically, evaluates how comparative genomics, cross-species functional conservation, and shared technical platforms enable synergistic improvement across all major cucurbits.

### 5.1. Disease Resistance Research Landscape Across Key Cucurbits

#### 5.1.1. Watermelon (*Citrullus lanatus*)

Research centers on *Fusarium* wilt (Fon), causing 30–80% yield loss. *Fom*-1 and *Fom*-2—cloned NLR genes—confer race-specific resistance to Fon 0 and 1 [74,75]. Race 2 resistance is polygenic, with QTLs mapped to chromosomes 9, 10, and 11 [76]. *ath-miR164a* targeting *Cla97C04G079030* (NAC TF) modulates Fon response [76]. Grafting onto bottle gourd (*Lagenaria siceraria*) or pumpkin rootstocks provides effective, non-genetic resistance to soil-borne pathogens [74,77]. However, PM and gummy stem blight resistance genetics remain underexplored [74,77].

#### 5.1.2. Melon (*Cucumis melo*)

Melon exhibits deeper resistance genetics than cucumber, particularly for PM. *Pm-w* (Chr5, CC-NLR) confers resistance to races 1 & 3 [78]; *CmPMRl*/*CmPMRs* (Chr12/10) and *Cmpmr2F* (Chr12, 26.25 kb) define race-1 and race-2F resistance [79,80]. *Cm-mlo38*/*Cm-mlo44* are validated S genes—VIGS knockdown enhances PM resistance [81]. DM and virus resistance genes (*Wmr*, *Prv*, *Vat*, *cmv1*, *nsv*) are also well-characterized [82,83].

#### 5.1.3. Pumpkin (*Cucurbita* spp.)

As a premier rootstock, pumpkin enhances scion resistance to PM and *Phytophthora* blight [77,84,85]. *PM*-0, introgressed from *Cucurbita okeechobeensis*, is deployed commercially and mapped to Chr3 (*C. moschata*) and Chr10 (*C. pepo*) [85,86]. *CpPM10.1* (RPW8-domain) underlies PM resistance in zucchini [87]. ToLCNDV resistance in *C. moschata* is monogenic and dominant [88,89,90]. Despite completed genome sequencing, systematic annotation of resistance genes lags [85,91].

### 5.2. Comparative Genomics: Conservation, Divergence, and Opportunity

#### 5.2.1. Evolution of Resistance Gene Families

Cucurbits share a distinctive resistance gene architecture: low NLR copy number (cucumber: approximately 60–70; melon: approximately 75; watermelon: approximately 55), near-complete absence of TNLs, high pseudogene content (approximately 30–50%), and predominant tandem clustering (60–70% of R genes) [39,92,93]. In contrast, regulatory families (RLK, WRKY, MYB) are highly conserved in size and structure [93,94]. This suggests evolutionary pressure to maintain compact, rapidly evolving sensor clusters while preserving stable signaling and transcriptional machinery.

#### 5.2.2. Collinearity and Synteny of Resistance Loci

Only a subset of R genes are orthologous across cucumber, melon, and watermelon; most show presence/absence (P/A) polymorphism, indicating frequent lineage-specific gene loss or rearrangement [93]. Orthologous MLO and WRKY genes exhibit high sequence conservation and functional equivalence—such as *Cm-mlo38* knockdown mirrors *CsMLO8* knockout in PM resistance [71,81]. Crucially, synteny analysis reveals conserved “resistance hotspots”: the *Pm5.1* region on cucumber Chr5 shows collinearity with PM QTLs in melon Chr5 and watermelon Chr4, suggesting a deeply conserved, functionally important locus amenable to cross-species marker transfer and editing [93,95].

### 5.3. Cross-Species Translation: Leveraging the Cucumber Model

#### 5.3.1. Functional Conservation Enables Direct Transfer

The *MLO* paradigm is universally applicable: loss-of-function edits in *Cm-mlo38*, *Cm-mlo44*, and *CpMLO* homologs all enhance PM resistance [71,81,87]. Similarly, *WRKY* TFs show conserved roles in SA/JA signaling across cucurbits and rice [96,97,98,99,100,101,102].

#### 5.3.2. Technical Platforms Are Highly Portable

GWAS successfully identified a major Begomovirus resistance QTL in pumpkin, explaining 60% of variance [86,103,104,105]. CRISPR editing of *ERECTA* homologs generated compact plant types in melon and watermelon; *ALS* editing conferred herbicide resistance in watermelon [106,107,108]. KASP markers developed for melon PM resistance are directly deployable in cucumber and pumpkin breeding [109,110,111].

#### 5.3.3. Wild Germplasm and Rootstock Systems Provide Complementary Leverage

Wild *Cucumis* species (*C. hystrix*, *C. amarus*) harbor novel resistance alleles introgressed into cultivated melon [91,112]. Pumpkin rootstocks not only provide physical barrier effects but also mediate systemic resistance—recent evidence shows graft-transmissible sRNAs modulate defense gene expression in scions [94,113,114]. This creates a powerful triad: (i) cucumber-derived molecular tools, (ii) melon/watermelon wild germplasm, and (iii) pumpkin rootstock-mediated immunity—enabling holistic, multi-layered resistance engineering.

## 6. Conclusions and Forward-Looking Perspectives

### 6.1. Synthesis of Core Advances

This review integrates three decades of research to present a unified framework for cucurbit disease resistance. First, resistance to PM, DM, and TLS is predominantly quantitative, with QTLs clustered in genomic “immune hubs”—notably Chr5 and Chr6—where NLR, MLO, and RLK genes form evolutionarily dynamic, functionally interlinked clusters. Second, molecular mechanisms have evolved from linear pathways to multi-layered networks: pathogen perception (RLK/NLR)—signal integration (NPR1/WRKY)—effector execution (PR/enzymes), with phenylpropanoid metabolism and ROS homeostasis forming the universal “defense chassis” Third, translational success is proven: *CsaMLO8* and *eIF4E* edits demonstrate that non-transgenic, durable resistance can be engineered without yield penalty—validating functional genomics as a breeding engine. Fourth, comparative genomics confirms that cucumber’s insights are broadly portable: conserved MLO function, synteny of resistance hotspots, and shared technical platforms enable coordinated improvement across the entire Cucurbitaceae.

### 6.2. Persistent Challenges

Despite progress, critical bottlenecks remain:

Genetic resolution: Many QTLs—especially for TLS—span >5 Mb with hundreds of genes; fine mapping is hampered by limited recombination and pathogen race complexity.

Mechanistic depth: Networks remain largely correlative; <10% of predicted TF–target interactions are experimentally validated; temporal/spatial resolution of omics data is insufficient to resolve cell-type–specific dynamics.

Translational friction: Regulatory uncertainty, lack of standardized phenotyping, and weak academia–industry feedback loops stall the movement of >99% of candidate genes from lab to field.

### 6.3. Strategic Priorities for the Next Decade

To accelerate impact, we propose three interdependent priorities:

Build translational infrastructure: Establish international, open-access “Cucurbit Resistance Hubs” integrating high-throughput phenotyping, genome editing, and field trial networks—standardizing protocols and sharing data.

Adopt predictive, network-guided breeding: Move beyond single-gene editing to engineer resilient immune circuits—such as simultaneously editing *CsaMLO8* and tuning *CsWRKY50* expression to balance resistance strength and fitness costs.

Forge cross-species breeding alliances: Create consortia linking cucumber genomicists, melon breeders, watermelon pathologists, and pumpkin rootstock specialists to co-develop pan-cucurbit resistance solutions—transforming fragmented efforts into unified, family-wide resilience.

## Figures and Tables

**Table 1:** Representative QTLs and major candidate genes associated with powdery mildew resistance in cucumber.

QTL/ Gene	Chromosome	Position (Mb/cM)	Variance Explained (R^2^)	Candidate Gene(s)/Type	Evidence Status	Inheritance	Reference
*Pm1.1*	Chr. 1	~41.1 kb region	18.40%	*Csa1M064780.1*, *Csa1M064790.1* (CRK genes)	Fine-mapped with candidate genes	Dominant	[15]
*pm5.1*	Chr. 5	~170 kb region	20.8–41.0%	MLO-like gene (*Cucsa.308270*/*Csa010846*)	Functionally validated/cloned	Recessive	[14]
*pm5.2*	Chr. 5	16.35–24.99 Mb (QTL-seq)	30–74.5%	*CsGy5G015660* (LRR RPK2); *CsMLO8* (S gene)	Functionally supported candidate region	Incomplete recessive/recessive	[10,16]
*pm6.1*	Chr. 6	11.01–12.42 Mb (QTL-seq)	11%	Not specified	Not resolved	-	[16]
*CsPM5.2*	Chr. 5	-	10.9–20.1%	*PHO1*;*H3* (phosphate transporter-like protein)	Functionally validated/cloned	Dominant	[17]

**Table 2:** Representative QTLs and major candidate genes associated with downy mildew resistance in cucumber.

Locus/ Gene	Chromosome	Position (Mb/cM)	Variance Explained (R^2^)	Inheritance	Candidate Gene(s)/Type	Evidence Status	Reference
*dm1.1*	Chr. 1	~4.26 Mbp	18.60%	Quantitative	NBS-type RGAs (predicted)	Candidate interval	[18]
*dm2.1*	Chr. 2	–	28.20%	Complete dominance	–	Candidate interval	[21,24]
*dm4.1*	Chr. 4	~1.2 Mbp	8–50.7%	Additive	*CsLRK10L2* (RLK)	Candidate interval with supported gene	[20,21,23]
*dm5.1*	Chr. 5	–	5–30%	Additive	NBS-type RGAs (predicted)	Candidate interval	[18,20,24]
*dm5.2*	Chr. 5	~13.7 MbpMb	10.7–27.2%	Quantitative	NBS-type RGAs (predicted)	Candidate interval	[18,21,24]
*dm5.3*	Chr. 5	~1.37 MbpMb	19.50%	Quantitative	*CsSIB1* (sigma factor-binding protein); NBS-type RGAs (predicted)	Candidate interval with supported gene	[18,25]
*CsaDMR6-2*	Chr. 5	–	–	Recessive	*DMR6* homolog	Functionally supported candidate	[26]

**Table 3:** Summary of molecular markers for resistance-related loci.

Resistance-Related Locus	Marker Type	Marker Name/Region	Co-Segregation/Accuracy	Scope	Breeding Application Status	Reference
*pm5.1*/*CsMLO8*	KASP	MLO8-KASP1 (exon1/exon5 deletion)	>98%	Broad-spectrum; multiple PM races	Widely used in commercial breeding	[63,64]
eIF4E (virus resistance)	CRISPR-mediated mutation detection	Mutation detection at gRNA1/gRNA2 target sites (1st & 3rd exons)	–	Broad-spectrum vs. viruses (CVYV, ZYMV, PRSV-W)	Applied in antiviral line development and large-scale production	[60,61]
*dm5.1*/*dm4.1* (DM resistance)	SSR, SNP	SSR03943, SSR19172, dm4.1, etc.	15–30% R^2^	Specific populations (e.g., PI 197088)	Used for QTL mapping and preliminary MAS	[71]
*fw2.1* (wilt resistance)	AFLP, SSR, InDel	E25M70, CSWCT06A, InDel1248093–InDel1817308, etc.	–	Specific populations (e.g., F2 from Superina/Rijiecheng derivatives)	Potential breeding target; under study	[71]

## Data Availability

All data generated or analyzed during this study are included in this published article.

## Appendix A

**Table A1:** Comparative features of PM, DM, and TLS QTLs.

Feature	Powdery Mildew, PM	Downy Mildew, DM	Target Leaf Spot, TLS
Number of studies	Many	Many	Few
Number of QTLs	Many	Numerous	Few
Major-effect QTLs	*Pm1.1*, *pm5.1*, *pm5.2*, *CsPM5.2*	*dm4.1*, *dm5.1*, *dm5.2*, *dm5.3*	*cca-3*, *gTLS5.1*, *gTLS5.2*, *gTLS7.1*
Cloned/fine-mapped genes	*CsMLO8* (*pm5.1*), *CsPM5.2*, *Pm1.1* (candidate), *pm5.2* (candidate)	*dm5.3* (candidate: *CsSIB1*), *dm4.1* (candidate: *CsLRK10L2*)	*cca-3* (candidate: *Csa6M375730.1*)
Inheritance	Dominant (*Pm1.1*, *CsPM5.2*); recessive (*pm5.1*, *pm5.2*)	Mostly quantitative; additive/dominant/epistatic effects	Recessive (*cca-3*)/quantitative (GWAS)
Hotspot chromosomes	Chr. 1, Chr. 5	Chr. 4, Chr. 5	Chr. 5, Chr. 6, Chr. 7

**Table A2:** Summary of cucumber PM GWAS studies.

Study	Sample Size	Genotyping Method	No. of SNPs	Significant Loci	Key Findings	Reference
Liu et al., 2020	109 core accessions	Resequencing	Not specified	18	Identified 18 loci (*dmG1.4*–*dmG7.2*); *dmG2.1* and *dmG7.1* were novel; predicted candidates include *Csa1G575030*, *Csa2G060360*, *Csa4G064680*, *Csa5G606470*, *Csa7G004020*, etc.	[115]
Liu et al., 2021	94 resequenced lines	Resequencing	Not specified	13	Identified 13 loci; *pmG2.1*, *pmG3.1*, *pmG4.1* were novel; strong signals at *pmG2.1*, *pmG5.2*, *pmG5.3*; implicated MLO genes, with *Csa5G622830* and *CsGy5G015660* as candidates.	[116]
Xu et al., 2024	299 accessions	Resequencing	1.8 M	50	Identified 50 significant loci associated with PM resistance and revealed parts of the molecular mechanism.	[46]
Nie et al., 2015	BC3F1 and BC2F2 populations	Segregating-population analysis	Not specified	1 (pm5.1)	Fine-mapped recessive PM resistance gene *pm5.1* to ~170 kb; identified an MLO-like gene as candidate.	[14]

**Table A3:** Characteristics of cucumber NLR genes.

Ype	Number	Chromosomal Distribution	No. of Tandem Clusters	Segmental Duplication Events	References
CNL	33–61	Chr1–7 (uneven)	16 (clustered)	Present	[40]
TNL	2	Chr. (not specified)	Few or none	Few	[40]
Total	57–63	Whole genome	–	–	[39,40]

**Table A4:** Cucumber genes with functional evidence (as listed).

Gene ID	Chromosome	Disease	Expression Change	Validation Method	Resistance Type	Reference
*CsMLO1*	Chr5	PM	Differential expression	Gene knockout	Recessive resistance (loss-of-function)	[14]
*Csa5G622830*	Chr5	PM	Differential expression	Homology-based annotation	Involved in resistance	[116]
*CsGy5G015660*	Chr5	PM	Differential expression	Homology-based annotation	Involved in resistance	[116]
(CNL gene, e.g., *Csa3G172400*)	Chr3	PM	Upregulated	qRT-PCR	Involved in resistance	[40]
(CNL gene, e.g., *Csa5G647590*)	Chr5	DM	Upregulated	qRT-PCR	Involved in resistance	[40]

**Table A5:** Candidate genes.

Gene ID	Functional Annotation	PM log_2_FC	DM log_2_FC	TLS log_2_FC	Network Degree	GO Significance
*Csa_4G370550*	Ethylene-responsive TF ERF014	2.910	4.852	1.909	0	0.00919
*Csa_4G641690*	Hypothetical protein	3.227	2.949	1.193	2	>0.05
*LOC101207158*	Cis-regulatory region–binding protein	2.451	5.809	−2.048	7	0.01493
*Csa_3G664560*	Hypothetical protein	Not significant	Not significant	4.659	0	GO:0015979

**Table A6:** Summary of multi-omics studies.

Study	Year	Disease/Treatment	Omics/Approaches	Key Findings	Data Availability (GEO/SRA)	Reference
Wang et al. (2017) (cited in Yu et al., 2019)	2017	TLS (*Corynespora cassiicola*)	Transcriptomics (RNA-seq)	Identified CsMLO genes involved in response to C. cassiicola	N/A	[65]
Li et al. (2018) (cited in Yu et al., 2019)	2018	TLS (*Corynespora cassiicola*)	iTRAQ proteomics + transcriptomics (RNA-seq)	Identified CsMLO genes involved in response to C. cassiicola	N/A	[65]
Yu et al. (2019)	2019	TLS (*Corynespora cassiicola*)	Small RNA-seq, qRT-PCR, VIGS, transient expression, biochemical assays	CsMLO1 and CsMLO2 are negative regulators; silencing activates Ca^2+^/ROS/ABA signaling, PR genes, and cell wall strengthening. miRNAs (miR164d, miR396b, Novel-miR1, Novel-miR7) negatively regulate resistance; targets (NAC, APE, 4CL, PAL) positively regulate resistance.	SRP117262 (mRNA), SRP117230 (small RNA)	[65]
Luan et al. (2019)	2019	DM (Pseudoperonospora cubensis)	Gene cloning, expression analysis, overexpression validation	CsWRKY50 mediates defense against P. cubensis via ROS and hormone signaling to enhance resistance.	N/A	[55]
Zhang et al. (2019)	2019	Propamocarb treatment (DM-related control)	Tag-Seq transcriptomics, qRT-PCR, biochemical assays, functional validation	CsMCF responds to propamocarb and biotic stress; overexpression reduces pesticide residue and enhances antioxidant capacity.	N/A	[117]

**Table A7:** Comparison of functional validation methods.

Method	Time Cost	Technical Difficulty	Reliability	Applicable Stage	No. of Cucumber Cases	Advantages	Limitations
Transgenic overexpression	6–12 months	High	High	Candidate gene validation	[56,57,58,59]	Stable inheritance; enables deep mechanistic study	Long cycle; high technical requirements; positional effects/gene silencing; overexpression of some genes may increase susceptibility
CRISPR knockout	4–8 months	Medium–high	High	Loss-of-function validation	[60,61,62,63,64]	Precise and efficient; enables non-transgenic resistant plants; potential for broad-spectrum resistance	Off-target risk; lower transformation efficiency in hard-to-regenerate genotypes
RNAi	6–10 months	Medium	Medium	Expression suppression	[66,67]	Reduces target gene expression; useful for gene dosage effects	Potential off-targets; efficiency/stability vary; hard to achieve complete silencing
VIGS	2–4 weeks	Low–medium	Medium	Rapid screening/preliminary validation	[57,65,68]	Fast, low-cost; suitable for high-throughput preliminary screening; no stable transformation required	Transient effect; sensitive to environment; not suitable for all plants/genes
Transient expression	1–2 weeks	Low	Low	Preliminary validation	[65,69]	Extremely fast and low-cost; suitable for in vitro/tissue-level tests	Transient; results need confirmation via stable transformation
